# Predictive value of the systemic immune inflammatory index in cardiac syndrome x

**DOI:** 10.1186/s12872-023-03157-3

**Published:** 2023-03-23

**Authors:** Yusuf Akın, Mehdi Karasu, Abdulmelik Deniz, Çetin Mirzaoğlu, Hasan Ata Bolayır

**Affiliations:** 1 Department of Cardiology, Fırat University Faculyt of Medicine, Elazıg, Turkey; 2 Department of Cardiology, Fethi Sekin Sehir Hastanesi, Elazıg, Turkey; 3 Department of Cardiology, Fatsa State Hospital, Ordu, Turkey; 4 Department of Cardiology, Malatya Turgut Ozal Universitesi Kardiyoloji ABD, Malatya, Turkey

**Keywords:** Cardiac syndrome x, Systemic immune-inflammation index, Predictiveness

## Abstract

**İntroduction:**

Patients with normal coronary arteries in whom increased vasospasm cannot be detected with the stress test should be evaluated in terms of cardiac syndrome x (CSX). İnflammatory systems are effective in endothelial activation and dysfunction in CSX. The systemic immune inflammation index (SII) is thought to be an important factor in determining the course of diseases, especially in infectious diseases or other diseases, as an indicator of the inflammation process. The aim of this study is to determine the role of SII levels in the diagnosis of CSX disease.

**Methods:**

The study group included 80 patients who applied to the cardiology department of Fırat University with typical anginal complaints between October 2021 and April 2022, and were diagnosed with ischemia after the myocardial perfusion scan, and then coronary angiography was performed and normal coronary arteries were observed.

**Results:**

When the study and control groups were examined according to age, gender and body mass index, hypertension, smoking, diabetes mellitus, dyslipidemia and family history, no statistical significant difference was observed between the groups. It was observed that there was a significant difference between the high sensitive C- reactive protin levels of the individuals in the study and control groups (*p* = 0.028). SII levels measured in samples taken from patients were significantly higher than control subjects (*p* = 0.003). SII cutoff at admission was 582 with 82% sensitivity and 84% specificity (area under the curve 0.972; 95% CI:0.95–0.98;*p* < 0.001).

**Conclusion:**

It has been demonstrated that systemic SII parameters, which can be simply calculated with the data obtained from the complete blood count and do not require additional costs, can contribute to the prediction of CSX disease.

## Introduction

In the angiography performed in the preliminary diagnosis of coronary artery diseases, 30% of the patients are reported as normal coronary arteries. However, it has been reported that patients with normal coronary arteries in whom increased vasospasm cannot be detected with the stress test should be evaluated in terms of cardiac syndrome x (CSX) [1].

CSX was defined by Kemp et al. (1973) in patients with chest pain, ischemic exercise electrocardiography(ECG) responses, and normal angiographic procedures performed on the coronary arteries. It has been reported that since there is no clear information about the underlying causes of chest pain in patients with normal coronary arteries in the coronary angiography procedure, they defined this patient group as 'Syndrome X' [2]. Although there is no clear information about the etiology of CSX disease, it is estimated that the etiology of the disease is caused by microvascular dysfunction [3].

CSX is a clinical picture especially seen in elderly men and postmenopausal women. While chest pain increasing with effort is observed in 20% of patients diagnosed with CSX, it is reported that the remaining patients have angina pectoris characterized by transient chest pain at rest and ST elevation on ECG [4].

It has been reported that women undergoing coronary angiography are classified as normal 3 times more than men, and 72% of CSX patients are postmenopausal women [5].

Although these patients are generally stable, chest pain is significant and limiting at certain intervals. For this reason, it is stated that although patients are benign in terms of survival, they have a significant adverse effect on quality of life [6].

It has been stated that patients diagnosed with CSX have pathophysiological abnormalities due to many factors. These abnormalities are reported to be caused by many factors, including abnormal coronary flow reserve, decreased insulin resistance, abnormal autonomic control, increased sodium-hydrogen level, abnormal cardiac sensitivity, and microvascular spasm (6).

Tousoulis et al. (2001) determined that inflammatory systems are effective in endothelial activation and dysfunction in CSX. It has been determined that blood levels of adhesion molecules synthesized by active endothelial cells due to inflammatory stimuli increase in patients with a diagnosis of CSX [7]. In addition, it was determined that increased high sensitivity C-reactive protein (hs-CRP) levels in these patients were associated with more severe angina pectoris and more active disease. It has been determined that CSX patients with increased hs-CRP levels have more frequent episodes of ST segment depression in Holter-ECG monitoring and earlier and more pronounced ischemic ST segment changes during exercise stress testing [8].

Myocardial perfusion scanning(MPS) is one of the most commonly used non-invasive test procedures in the evaluation of known or suspected coronary artery disease (CAD) [9]. In recent years, new strategies have been increasingly advocated and investigated to reduce radiation exposure, reduce costs, and increase laboratory efficiency [10]. For this reason, the importance of diagnostic tests that will reduce the need for nuclear imaging on the path to differential diagnosis has increased.

The systemic immune inflammation index (SII) is thought to be an important factor in determining the course of diseases, especially in infectious diseases or other diseases, as an indicator of the inflammation process [11]. It is stated that the SII is a new generation inflammation biomarker created with whole blood parameters. This biomarker is the result of the product of the neutrophil count and platelet count divided by the lymphocyte count [12].

The aim of our study is to determine the role of SII levels in the diagnosis of CSX disease, which has been shown in many previous studies to be closely related to important cardiovascular events such as CAD, heart failure (HF) and hypertension (HT).

## Methods

### Study design and measurements

The study group included 80 patients who applied to the cardiology department of Fırat University with typical anginal complaints between October 2021 and April 2022, and were diagnosed with ischemia after the MPS, and then coronary angiography (CAG) was performed and normal coronary arteries were observed. In the control group, a total of 80 volunteers with similar demographic characteristics, who came to the outpatient clinic for control purposes and whose cardiovascular stress test was evaluated as negative, were included. Patients in control group did not have active cardiac complaints as a result of the evaluation of another physician who was unaware of the study. The cardiovascular stress test were evaluated by another physician who was unaware of the first physician's evaluation and the study. Presence of unstable angina, history of previous myocardial infarction (MI), coronary vasospasm, moderate to severe heart valve disease, renal dysfunction(SCr > 1.5 mg/dL in men and > 1.4 mg/dL in women), hepatic dysfunction(more than double the upper limit of the lab ref.), presence of left ventricular systolic dysfunction(EF < 0.40), presence of malignant tumors, hematological disease, history of chronic or acute infection, and patients receiving steroid treatment were excluded from the study.

The diagnosis of HT was based on systolic blood pressure > 140 mm Hg, diastolic blood pressure > 90 mm Hg, or a history of antihypertensive drug use. Type 2 Diabetes mellitus (DM) was defined as the use of antidiabetic drugs or fasting blood sugar ≥ 126 mg/dl. Smoking history was defined as regular tobacco use. Participants in the patient and control groups did not have any antiischemic, antiarrhythmic, antiplatelet, statin and/or anticoagulant use.

### Myocardial perfusion scan (MPS)

Daily exercise approach or stress test (dipyridamole Tc-99 m MIBI protocol) was determined for MPS according to the standards determined by the Turkish Nuclear Society Working Group. The criteria set in this study were followed. For the stress test, the condition of 'no food or drink' for at least 4 h was determined. İf any calcium channel blocker or beta blocker drugs were discontinued 48 h before so that they did not cause a difference in heart rate or blood pressure and did not create any contraindications.

In the stress/rest test according to the modified Bruce protocol, 8–10 mCi was applied first, then 22–25 mCi. Target heart rate was calculated with the formula (220-age) × 0.85. The procedure was terminated when a situation that would constitute a contraindication for the continuation of the test developed in the patient during the effort. Intravenous administration of dipyridamole (0.14 mg/kg/min × 4 min) followed by Tc99m sestamibi (8–10 mCi as a stress dose) was performed when the patient reached heart rate (0.85 × peak) and above and developed clinical weakness for the treadmill. It was applied with 22 to 25 mCi (approximately 3 × 8–10 mCi) from the 30th minute of the protocol; Tc 99 m sestamibi application was repeated at the end of the resting phase, approximately three hours later. Finally, MPS was performed after 45–60 min.

### Coronary angiography(CAG)

The standard Judkins method was used during CAG, a femoral or radial catheter was used for CAG, and the test results were blindly examined by the person who performed two different angiography protocols. The channel blockers adenosine, nitroglycerin or calcium were not administered. Hyperventilation testing was performed to detect arterial spasm and exclude diagnosed patients. Coronary arteries were considered abnormal when illuminated narrowing or irregularity was detected.

### Laboratory measurements

Blood samples were taken from peripheral veins of patients who fasted overnight. Auto-analyzers were used to evaluate hematological results. Abbott Cell-Dyn 3700 device was used for total or differentiated leukocyte counts. Abbott Architect C16000 automated analyzer (Abbott Lab, Abbott Park, IL, USA) was used for total and specific high-density lipoprotein (HDL) cholesterol, triglycerides and fasting blood glucose levels. The Friedewald equation was used to calculate serum low-density lipoprotein (LDL) cholesterol concentrations. The participants in the patient group applied to the hospital in the early period after the onset of symptoms, and their laboratory parameters were checked on the day of the mps recording. The time between MPS and symptom onset was short. The laboratory parameters of the participants in the control group were checked at the time of application and cardiovascular stress test was performed on the same day.

### Statistical analysis

SPSS for Windows v 20.0 (SPSS, Chicago, IL, USA) program was used for statistical analysis. Descriptive statistical analyzes such as mean, standard deviation, rate and frequency data were performed. The validity of the normal distribution for permanent variables was determined using the Kolmogorov–Smirnov test. Student t-test and Mann–Whitney U test were used for parametric and non-parametric data, respectively. Intergroup comparisons were made for certain variables with the chi-square test. In addition, Pearson correlation analysis was performed with the established logistic regression model to find the effects of the parameters. With the calculation of the standard beta coefficient, *p* < 0.05 was considered statistically significant at the 95% CI confidence interval.

## Results

Table 1 shows the baseline clinical and demographic characteristics of the Study population. When the study and control groups were examined according to age, gender and body mass index (BMİ), HT, smoking, DM, dyslipidemia and family history, no statistical significant difference was observed between the groups.

**Table 1:** Main characteristics of study groups (*n* = 160**)**

Parameters	Control group (*n* = 80)	Patients with CSX (*n* = 80)	P
Age [years]	52.9 ± 9.5	54.6 ± 8.9	0,566
BMİ [kg / m2]	27.5 ± 3.3	27.3 ± 4.0	0,752
Woman	32 (40.0%)	33 (42.0%)	0,853
DM	12 (15.0%)	10 (12.5%)	0,503
HT	20 (25.0%)	18 (22.5%)	0,745
Dyslipidemia	20 (25.0%)	18 (36.7%)	0,745
family history	7 (8.75%)	10 (12.5%)	0,206
Smoking	12 (15.0%)	10 (12.5%)	0,745

Data are given as mean ± standard deviation or number (percent), *CSX* Cardiac syndrome x

Table 2 shows the laboratory results of the study groups. It was observed that there was a significant difference between the hs-CRP levels of the individuals in the study and control groups (*p* = 0.028). SII levels measured in samples taken from patients were significantly higher than control subjects (*p* = 0.003).

**Table 2:** Laboratory Results of Study Groups

Parameters	Control group (*n* = 80)	Patients with CSX (*n* = 80)	P
Glucose [mg/dL]	118.4 ± 44.1	124.1 ± 59.7	0,490
Creatinine [mg/dL]	0.88 ± 0.2	1.00 ± 0.4	0,266
Uric acid [mg/dL]	6.8 ± 2.1	6.2 ± 1.7	0,580
WBC count [103/mm3]	9.8 ± 2.4	10.3 ± 2.6	0,269
Hemoglobin [g/dL]	13.4 ± 1.7	13.7 ± 1.5	0,255
Platelet count [10 [10³/mm³]	242.4 ± 62.4	278.2 ± 56.8	0,171
Total cholesterol [mg/dL]	172.0 ± 79.6	180.1 ± 77.4	0,615
Triglyceride [mg/dL]	124.0 (80.0–190.0)	123.5 (78.25–161.25)	0,683
LDL-cholesterol [mg/dL]	113.1 ± 57.3	116.0 ± 58.7	0,790
HDL-cholesterol [mg/dL]	41.0 (33.5–48.0)	43.5 (35.0–49.0)	0,820
hs-CRP [mg/L]	3.0 (1.1–4.7)	4.8 (2.6- 6.6)	0,028
NLR	1.52 (1.38–1.77)	1.82 (1.66–2.01)	0,084
LVEF [%]	60.0 ± 4.9	58.2 ± 5.1	0,442
SII	388 (309–477)	611 (542–704)	0,003

Data are given as mean ± standard deviation, number (percentage), or median (interquartile range), *HDL* High-density lipoprotein, *hs-CRP*-high-sensitivity C-reactive protein, *LDL* Low density lipoprotein, *LVEF* Left ventricular ejection fraction, *CSX* cardiac syndrome X, *WBC* Leukocyte, *SII* İndex of systemic immune inflammation, *NLR* Neutrophil / lymphocyte ratio

After univariate logistic regression analysis to reveal possible causes of CSX, there was a statistical significant correlation between hs-CRP and SII parameters and CSX. Following multiple linear regression analysis, a high SII level was able to predict CSX disease (OR = 0.982; 95% CI:0.969–0.995; *p* = 0.005) (Table 3).

**Table 3:** Univariate and multiple linear regression analysis showing predictors for CSX

Variables	Univariate (95% Cl)	P	Multivariate (95% Cl)	P
hs-CRP	1.127 (1.008–1.261)	0,036	1.099 (0.982–1.230)	0,100
SII	0.980 (0.967–0.992)	0,002	0.982 (0.969–0.995)	0,005

*CI* Confidence interval, *SII* İndex of systemic immune inflammation, *hs-CRP* High-sensitivity C-reactive protein, *OR* Odds ratio

**Fig. 1:**
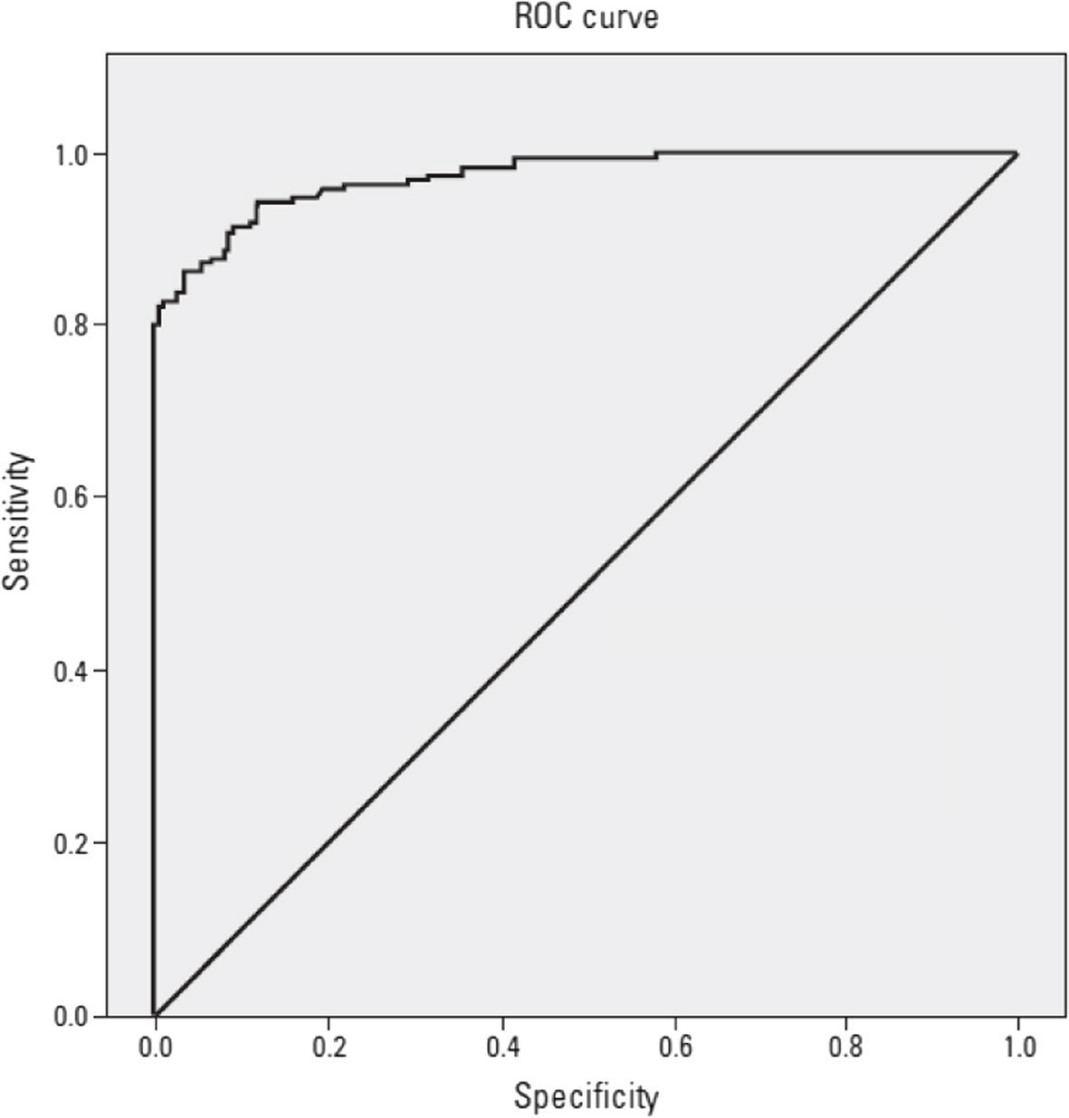
Receiver operating characteristic (ROC) curve of the SII parameter for the diagnosis of CSX. SII cutoff at admission was 582 with 82% sensitivity and 84% specificity (area under the curve 0.972; 95% CI:0.95-0.98; *p*< 0.001). SII: Systemic immune-inflammation index

To predict the presence of CSX in all subjects included in the study population on the basis of receiver operating characteristic (ROC) curve analysis, the SII cutoff at admission was 582 with 82% sensitivity and 84% specificity (area under the curve 0.972; 95% CI:0.95–0.98;*p* < 0.001) (Fig. 1).

## Discussion

As a result of our observations, our study is the first to examine the relationship between CSX and SII in MPS positive patients. It has been demonstrated that SII parameters, which can be simply calculated with the data obtained from the complete blood count and do not require additional costs, can contribute to the prediction of CSX disease.

It is thought that chronic inflammation has an effect on the formation of many important diseases such as cancer, cardiovascular disease [13], DM and metabolic syndrome [14–17]. It has been reported that inflammatory parameters increase in pulmonary embolism, acute renal failure, pulmonary arterial hypertension, peripheral arterial disease and cerebrovascular diseases in which endothelial dysfunction and inflammation play a role [18]. Recent studies in the literature have reported an increase in inflammatory parameters in CAD, HF, and acute MI [18, 19]. Again, in many studies, it has been observed that there is a strong relationship between endothelial dysfunction and systemic immunoinflammation [20].

Neutrophil / lymphocyte ratio (NLR) has been reported to be an independent predictor of cardiovascular events and mortality in ST-elevation MI [21]. It has also been reported that platelet /lymphocyte ratio (PLR) is an effective marker for severe atherosclerosis [13]. Li et al. showed that interleukin-6 and hs-CRP were significantly higher in patients with coronary artery ectasia(CAE) compared to patients with normal coronary arteries [22].

Recio-Mayoral A et al. showed that systemic inflammation plays an important role in CSX by causing microvascular dysfunction [23]. Okyay K. et al. showed that patients with CSX have high NLR levels, and that inflammation may be associated with impaired myocardial perfusion in these patients [24]. It has been shown that monocyte / HDL ratio and mean platelet volume, which are other markers of inflammation, increase in CSX patients [25, 26]. In a study conducted by Bozcali et al. (2014) to evaluate Galectin-3 serum concentrations in patients with a diagnosis of CSX, it was reported that the level of hs-CRP increased in individuals with CSX [27]. Although the hs-CRP levels of the individuals in the CSX group were higher than the hs-CRP levels of the individuals in the control group in our study, no significant difference was observed in the NLR levels between the two groups.

It is stated that SII is associated with markers such as NLR, PLR, and monocyte-lymphocyte ratio, which are determined as parameters of the SII [28, 29]. Several studies have suggested that SII may more comprehensively represent the inflammatory state compared with NLR as well as neutrophil and lymphocyte counts [30, 31].

Hu et al. first reported that SII was used in hepatocellular carcinoma [32]. First, Seo et al. reported the predictive value of SII in patients with chronic HF [33]. Esenboğa et al. (2022) determined the SII in the study in which individuals with isolated CAE, obstructive coronary artery disease and normal coronary anatomy were compared; high SII levels have been shown to be associated with CAE and occlusive coronary disease [34].

In the studies of Ya-Ling Yang et al. (2020) investigating the relationship between SII and clinical outcomes of CAD; SII has been shown to be more effective than traditional methods in predicting major cardiovascular events in patients undergoing coronary intervention [35]. In a recent study on this subject, Yaşar E. et al. revealed that there is a significant relationship between SII and microvascular dysfunction in patients with CSX.(18) In our study, unlike this study, only patients diagnosed with ischemia by MPS were included and compared with a completely normal control group in terms of symptoms and signs. Thus, it was tried to determine a cutoff value that would not require advanced imaging methods by providing a more sensitive comparison opportunity.

In our study, it was shown that SII is a parameter that can predict CSX disease in which inflammation is effective in its etiology. To predict the presence of CSX, the SII threshold at admission was 582 with 82% sensitivity and 84% specificity.

## Conclusions

As a result of our observations, our study is the first to examine the relationship between CSX and SII in MPS positive patients. It has been demonstrated that SII parameters, which can be simply calculated with the data obtained from the complete blood count and do not require additional costs, can contribute to the prediction of CSX disease. However, this relationship needs to be confirmed by future studies with larger populations and prospective studies in this area.

### Limitations

Our study has some limitations. First, it was a single-center, retrospective cross-sectional study and included a relatively limited number of patients. Second, we could not evaluate the real plaque load in patients without evidence of lumen narrowing on angiography, as there may be plaque load on the coronary vessel walls as well.

## Data Availability

All needed data can be obtained from corresponding author.
